# Aspirin Actions in Treatment of NSAID-Exacerbated Respiratory Disease

**DOI:** 10.3389/fimmu.2021.695815

**Published:** 2021-06-25

**Authors:** Esha Sehanobish, Mohammad Asad, Mali Barbi, Steven A. Porcelli, Elina Jerschow

**Affiliations:** ^1^ Department of Medicine, Albert Einstein College of Medicine, Bronx, NY, United States; ^2^ Department of Microbiology and Immunology, Albert Einstein College of Medicine, Bronx, NY, United States

**Keywords:** N-ERD, aspirin, COX-1 inhibitor, NSAIDs, nasal polyps, prostaglandins, leukotrienes, lipoxins

## Abstract

Non-steroidal Anti-inflammatory drugs (NSAID)-exacerbated respiratory disease (N-ERD) is characterized by nasal polyposis, chronic rhinosinusitis, adult-onset asthma and hypersensitive reactions to cyclooxygenase-1 (COX-1) inhibitors. Among the available treatments for this disease, a combination of endoscopic sinus surgery followed by aspirin desensitization and aspirin maintenance therapy has been an effective approach. Studies have shown that long-term aspirin maintenance therapy can reduce the rate of nasal polyp recurrence in patients with N-ERD. However, the exact mechanism by which aspirin can both trigger and suppress airway disease in N-ERD remains poorly understood. In this review, we summarize current knowledge of aspirin effects in N-ERD, cardiovascular disease, and cancer, and consider potential mechanistic pathways accounting for the effects of aspirin in N-ERD.

## Introduction

NSAID Exacerbated Respiratory Disease (N-ERD) is characterized by nasal polyp formation, asthma, and hypersensitivity to all cyclooxygenase-1 (COX-1) inhibitors, which are commonly used non-steroidal anti-inflammatory drugs (NSAIDs). COX-1 catalyzes the production of prostaglandins, thromboxanes and prostacyclins from arachidonic acid, and NSAIDs act by blocking the ability of COX-1 to initiate the biosynthesis of these mediators by catalyzing oxidation of arachidonic acid (1). The earliest known description of N-ERD in the medical literature can be traced to a 1922 article by Widal et al. (2) Over four decades later, Samter and Beers provided a more systematic characterization of the combination of aspirin-intolerance with nasal polyps and asthma, referred to initially as Samter’s triad (3, 4). Subsequently, this became known as aspirin exacerbated respiratory disease (AERD), and more recently as N-ERD to more accurately reflect its association with hypersensitivity to all drugs that inhibit COX-1 (5, 6). Clinically, N-ERD is characterized by an adult onset of severe nasal congestion followed by chronic rhinosinusitis, and eventually by the development of nasal polyps. Asthma is frequently although not universally present in N-ERD (7). Nasal polyps, which are benign growths in the paranasal sinuses that can obstruct airflow leading to difficulty in breathing and loss of olfactory sense, are a key feature of N-ERD, and the severity of sinonasal symptoms due to nasal polyps is often correlated with the severity of asthma symptoms (8). In addition, endoscopic sinus surgery decreases or abolishes reactions to aspirin in most N-ERD patients, and improves long term responses to aspirin desensitization and maintenance treatment (9, 10).

In N-ERD, the underlying inflammatory process of the upper and lower respiratory systems begins and occurs independently of the NSAID consumption. However, intake of COX-1 inhibitors triggers symptoms resembling an allergic reaction or an asthma attack (11). Around 15% of patients with N-ERD may be unaware of their hypersensitivity to these drugs (12). Surveillance studies indicate that N-ERD is not a rare condition. From one study by Rajan et al. it was found that approximately 7% of all adult asthmatics have N-ERD (13). The prevalence of N-ERD is higher in severe asthma patients (15%) followed by patients with nasal polyps (10%) (13). N-ERD patients have greater morbidity than aspirin tolerant asthma patients, as characterized by more frequent corticosteroid bursts, increased hospitalizations and emergency visits, and lower baseline forced expiratory volume in 1 second (FEV1) (12, 14).

Among treatment approaches that have shown benefit in N-ERD, aspirin desensitization followed by a continuous long-term aspirin administration has yielded significant improvement in the clinical course of many patients (15–19). This treatment reduces asthma and sinonasal symptoms, daily use of corticosteroids, ER visits or hospitalization due to asthma, and sinus infections (20, 21). Desensitization and aspirin maintenance therapy reduces and delays the occurrence of nasal polyps in more than 70% of N-ERD patients (22, 23). Even though the beneficial effects of aspirin desensitization and maintenance therapy are now accepted by many practitioners, the mechanisms, by which aspirin leads to the suppression of clinical symptoms and to polyp prevention are not well understood. In this review we focus on pathways by which aspirin can exert anti-inflammatory effects and prevent the recurrence of the nasal polyps in N-ERD.

## Immunological Features of N-ERD

N-ERD is characterized by increased numbers of eosinophils and mast cells, upregulation of type-2 (T2) pro-inflammatory cytokines, and abnormalities in the production of cysteinyl leukotrienes and prostaglandins (24–29). Hypersensitivity reactions are caused by inhibition of COX-1 by the NSAIDs. Even though increases in several inflammatory mediators such as cysteinyl leukotrienes and prostaglandins are associated with N-ERD, the exact events that result in the initiation and the progression of the disease are not yet known.

### Involvement of Leukotrienes and Their Receptors in N-ERD

5-lipoxygenase (5-LO) mediated oxidation of arachidonic acid produces cysteinyl leukotrienes (cysLTs) and leukotriene B_4_ (LTB_4_).

### CysLTs

Leukotrienes are a family of inflammatory lipid mediators that are synthesized from arachidonic acid by eosinophils, mast cells, macrophages, neutrophils and basophils (30–33). Leukotrienes C_4_ (LTC_4_), D_4_ (LTD_4_) and E_4_ (LTE_4_) are collectively known as cysLTs due to the presence of a cysteine residue. In neutrophils, monocytes, eosinophils, mast cells, and basophils, arachidonic acid is oxidized by 5-lipoxygenase (5-LO) to produce an unstable metabolite, leukotriene A_4_ (LTA_4_) (34). In monocytes, mast cells, eosinophils, and basophils, LTA_4_ upon conjugation with glutathione is then converted to LTC_4_ by leukotriene C_4_ synthase (LTC_4_S) (35–37). LTC_4_ is then extracellularly converted to LTD_4_ and its stable metabolite, LTE_4_ (38–40). LTC_4_, LTD_4_ and LTE_4_ are potent bronchoconstrictors in human and in other species (41–44). LTD_4_ functions *via* its interaction with a G protein-coupled receptor, cysteinyl leukotriene receptor 1 (CysLT_1_R), which has a lower affinity for LTC_4_ and LTE_4_. In addition, LTD_4_ and LTE_4_ have a similar affinity to another leukotriene receptor, cysteinyl leukotriene receptor 2 (CysLT_2_R), also a G-protein coupled receptor. There may also be other less investigated cysLT receptors that could be involved in the action of these lipid mediators (45, 46).

The over-production of cysteinyl leukotrienes is a characteristic feature of N-ERD (26, 35). Urinary levels of LTE_4_ are elevated at baseline and increase several fold after aspirin challenge in N-ERD patients compared to aspirin-tolerant asthma patients (47, 48). Several possible mechanisms have been proposed to account for the overproduction of cysLTs. For example, eosinophils and mast cells can produce cysLTs, raising the possibility that the overproduction of cysLTs is a result of the higher numbers or an increased activation of these cells in respiratory tissue of N-ERD patients (24, 25). The two main enzymes involved in the production of these lipid mediators are 5-LO and LTC_4_S. In case of biopsies from N-ERD patients, the percentages of mast cells and eosinophils staining positive for 5-LO was much higher than in the biopsies from the aspirin tolerant patients (36, 49). Platelets too can express LTC_4_S, and upon adhering to leukocytes they are capable of using the leukocyte-derived LTA_4_ as a substrate for conversion to LTC_4_. In this regard, it is interesting that N-ERD patients had a higher percentage of platelet-adherent leukocytes than aspirin-tolerant asthma patients (35). Another reason for the overproduction of cysLTs is related to the decrease in the prostaglandin E_2_ (PGE_2_) levels in nasal tissue samples including nasal polyps. Low PGE_2_ levels may contribute to the shift of the arachidonic acid metabolism pathway towards leukotriene synthesis. PGE_2_ inhibits cysLT production in people with N-ERD *via* the inhibition of 5-LO (50, 51). Therefore, the overexpression of cysLTs in N-ERD is likely a result of an increased activity of 5-LO and LTC_4_S, and an increased availability of the LTC_4_S due to the increase of platelet-adherent leukocytes (52).

Overexpression of cysteinyl leukotriene receptor CysLT_1_R in nasal biopsies of N-ERD patients has also been reported (53). The mRNA and the protein expression of CysLT_1_R can be increased by interleukin-13 (IL-13) and inteleukin-4 (IL-4), as observed in human monocytes and monocyte-derived macrophages (54). Both of these cytokines have an increased expression in N-ERD, suggesting that they could act as important drivers of pathology in N-ERD at least in part by augmenting cysLT receptor expression. Potentially, genetic polymorphisms in cysLT receptor genes could contribute to this mechanism of disease pathogenesis, although such polymorphisms have not yet been described in N-ERD. Little is currently known about the involvement of CysLT_2_R in N-ERD.

### LTB_4_

5-LO catalyzes the conversion of arachidonic acid to LTA_4_ (34). LTA_4_ can then be converted either to cysLTs as mentioned in the above section or can be converted to LTB_4_ by LTA_4_ hydrolase in neutrophils and eosinophils (55). LTB_4_ is a pro-inflammatory lipid mediator that functions as chemoattractant for eosinophils, monocytes, macrophages, and neutrophils (56–58). LTB_4_ can activate leukocytes *via* its interaction with a G-protein-coupled surface receptor BLT_1_ (59). LTB_4_ concentrations are elevated in the bronchoalveolar lavage fluid from patients with severe asthma, with eosinophils being the major source of LTB_4_ production (60). In N-ERD, along with an increase in the urinary level of the cysLT, LTE_4_, there is an increase in the urinary levels of an LTB_4_ metabolite during reactions to COX-1 inhibitions (61).

### Involvement of Prostaglandins and Their Receptors in N-ERD

Prostaglandins are lipid molecules derived from arachidonic acid by the action of the COX enzymes *via* endoperoxide intermediates such as prostaglandin G_2_ (PGG_2_) and H_2_ (PGH_2_). The latter leads to the production of the five main bioactive prostaglandins, prostaglandins D_2_ (PGD_2_), E_2_ (PGE_2_), F_2α_ (PGF_2α_), prostacyclin (PGI_2_) and thromboxane A_2_ (TXA_2_) (62). Three of these (PGD_2_, PGE_2_ and TXA_2_) have been implicated in the pathogenesis of N-ERD.

### PGD_2_

PGD_2_ is the main product of COX-derived intermediates in mast cells (63). It is also produced by eosinophils, although the lower expressions of the terminal PGD_2_ synthase gene compared to mast cells makes eosinophils a smaller source of this prostaglandin (64). PGD_2_ performs a wide range of functions through its interaction with its receptors: the thromboxane prostanoid (TP) receptors, and D-prostanoid (DP) receptors DP1 and DP2. The DP2 receptor is also known as chemoattractant receptor-homologous molecule expressed on T_H_2 cells (CRTH2). Upon binding to the TP receptor, PGD_2_ can function as a bronchoconstrictor and can also function as a potent chemoattractant for eosinophils and basophils *via* its interaction with CRTH2 (65–67). PGD_2_ can mediate vasorelaxation, inhibition of platelet aggregation, and bronchodilation primarily through stimulation of the DP1 receptor (68), and its signaling through this receptor also exerts an anti-inflammatory role in allergic inflammation (69). However, the functions of the DP1 and DP2 receptors in allergic inflammation are considered to be antagonistic (70), with DP1 stimulation resulting in mostly anti-inflammatory effects such as inhibition of cell migration, vasodilation, eosinophil apoptosis (71), whereas DP2 triggers pro-inflammatory effects by upregulating type 2 cytokines in Th2 cells (72, 73).

N-ERD is characterized by an overexpression of PGD_2_, as shown by the higher concentration of PGD_2_ in the sputum of N-ERD patients compared to aspirin tolerant asthma patients (74). N-ERD patients who have higher baseline levels of PGD_2_-metabolite in urine experience more severe clinical reactions to aspirin (75, 76), and the inability to tolerate aspirin desensitization has been attributed to the high levels of urinary PGD-metabolite (75). There are various mechanisms responsible for the high expression of PGD_2_ in N-ERD. Nasal polyps of N-ERD patients have a high concentration of mast cells and eosinophils which both express hematopoietic prostaglandin D synthase required for the synthesis of the PGD_2_ (64, 77, 78). Nasal polyp tissue and bronchial mucosa of asthmatic patients have been found to be rich in the cytokine thymic stromal lymphopoietin (TSLP), which can stimulate PGD_2_ production by mast cells (79). Nasal polyps have increased expression of TSLP compared to healthy nasal tissues (80–82), and Buchheit et al. showed that the levels of TSLP were significantly higher in the nasal polyps of N-ERD patients compared to nasal polyps of aspirin-tolerant asthma patients (64). Additionally in N-ERD patients, the levels of PGD_2_ paradoxically increase in plasma and urine after aspirin challenge, and correlate with the severity of the reaction to aspirin (64, 76, 83, 84). This is counterintuitive, considering that aspirin inhibits COX enzymes that represent a key step in prostaglandin synthesis, and could indicate an alternate pathway for PGD_2_ synthesis. Alternatively, this elevation of PGD_2_ could reflect effects of aspirin on targets other than COX enzymes, or indirect effects resulting from increased levels of leukotrienes (85).

### PGE_2_

In contrast to PGD_2_, the levels of PGE_2_ in N-ERD are greatly reduced both in peripheral blood cells and nasal tissue samples, including nasal polyps (86, 87). There are 4 subtypes of the E-prostanoid (EP) receptor to which PGE_2_ binds, designated as EP_1_, EP_2_, EP_3_ and EP_4_ (88, 89). PGE_2_ can function both as a pro- and an anti-inflammatory mediator. PGE_2_ may exhibit opposing functions *via* interaction with different EP receptors based on the cell type and the location (90). The pro-inflammatory effects of PGE_2_ is usually observed in various inflammatory conditions such as arthritis, inflammatory bowel disease, and also in different types of cancers (91–94).However, PGE_2_ exerts anti-inflammatory and bronchoprotective effects in the airways by suppressing allergen induced-inflammatory responses (95, 96). In N-ERD, PGE_2_ functions as an anti-inflammatory mediator as it prevents both airway obstruction and the increase in the urinary LTE_4_ associated with aspirin challenge in N-ERD (50, 97). PGE_2_ exerts its anti-inflammatory effects in the airways through the EP_2_ and EP_4_ receptors by activating protein kinase A (PKA). PGE_2_ binds to EP_2_ and EP_4_ receptors that activate adenylate cyclase. Next adenylate cyclase increases the levels of cellular cyclic AMP. This in turn activates PKA (98). PKA then phosphorylates 5-LO, thus directly inhibiting the catalytic activity of 5-LO and regulating the leukotriene synthesis by working as a brake for the production of cysLT (51). PGE_2_ signaling through its EP_2_ receptor blocks mast cell degranulation, limits eosinophil migration, and inhibits the allergen-stimulated release of mast cell-derived inflammatory mediators including PGD_2_ in the airways of asthma patients, providing another possible mechanism for its bronchoprotective action (99, 100).

The reduced expression and function of PGE_2_ in N-ERD could be attributed to a combination of factors. One of the enzymes responsible for the synthesis of PGE_2_ is COX-2, and expression of the mRNA for this enzyme is markedly reduced in nasal polyps of N-ERD patients compared to polyps and normal mucosa of aspirin-tolerant asthma patients (101). There is also diminished expression of EP_2_ receptors on mast cells and nasal fibroblasts of N-ERD patients (102, 103). Since PGE_2_ exerts its anti-inflammatory activities mainly through EP_2_, a reduced expression of the latter could decrease the ability of PGE_2_ to perform its anti-inflammatory actions in N-ERD patients. The reduction in PGE_2_ observed in N-ERD may also account at least in part for the increased baseline levels of PGD_2_ that are generally observed in N-ERD. Consistent with this, an elevated PGD_2_/PGE_2_ ratio in the nasal polyps of patients with chronic rhinosinusitis is often indicative of N-ERD (104). In addition, a reduction in the level of PGE_2_ in the nasal polyps of N-ERD patients may remove the normal brake on cysLT production, leading to the increased levels of leukotrienes (89). Consistent with the proposed important role of PGE_2_ deficiency in N-ERD, studies have shown inhaled PGE_2_ to serve as a bronchodilator in N-ERD patients (50, 105).

### TXA_2_

TXA_2_ is the main COX-product derived from platelets (106). It is a potent unstable vasoconstrictor and hydrolyzes to an inactive but stable form, thromboxane B_2_ (TXB_2_) (107). TXA_2_ is a pro-inflammatory prostanoid involved in platelet aggregation and activation, and facilitates leukocyte recruitment (108, 109). Studies in animal models show that TXA_2_ can function as a potent bronchoconstrictor and mediates its effects through interaction with the TP receptors (110–112). Similar to PGD_2_, an overexpression of TXA_2_ is also associated with the pathogenesis of N-ERD. The basal urinary level of the stable thromboxane metabolite (TX-M) is found to be higher in N-ERD patients espcially those who are unable to tolerate aspirin desensitization as a treatment (75). The exact mechanism for the overexpression of TXA_2_ is not known.

### Involvement of Lipoxins in N-ERD

In 1984, Serhan et al. first described a new set of oxygenated derivatives of arachidonic acid isolated from human leukocytes that were different from the other eicosanoids as they contained a conjugated tetraene structure (113). In a follow-up study, these trihydroxytetraenes, generated from the interactions of the 5- and 15-lipoxygenase (5-LO and 15-LO) pathways of human leukocytes, were designated lipoxins and considered to be a newly recognized series of arachidonic acid metabolites. The two main products were designated lipoxin A_4_ and lipoxin B_4_ (114). There are two major pathways for lipoxin biosynthesis. One of these involves platelet-leukocyte interactions in which leukocyte-produced LTA_4_ is converted to lipoxin A_4_ and lipoxin B_4_, by platelet 12-lipoxygenase (115). The other pathway involves the action of 15-LO on arachidonic acid to produce 15(S)-hydroxyeicosatetraenoic acid (15(S)-HETE) which is then used as a substrate by 5-LO to produce lipoxins (116). 5-LO is involved in the production of both leukotrienes and lipoxins, and is thus an integral part of both pro- and anti-inflammatory pathways.

Lipoxins are produced by a variety of cells, including airway epithelium, platelets and eosinophils, and they perform functions different than CysLTs (117, 118). Unlike CysLTs which mainly function as bronchoconstrictors, lipoxins exert anti-inflammatory properties and inhibit bronchoconstriction (119). Lipoxins inhibit eosinophilic and neutrophilic migration dampening the airway hyperreactivity and allergic airway inflammation (120–122). The anti-inflammatory effects of lipoxins are mediated by interaction with a high-affinity, G-protein-coupled receptor called ALX/FPR2 (lipoxin receptor (ALX)/N-formyl peptide receptor (FPR)-2) (120, 123–125). These lipid mediators bind to ALX/FPR2 present on T and B cells, and regulate B and T cell-mediated responses during resolution of inflammation (126, 127). ALX/FPR2 receptors are also found on natural killer and innate lymphoid type 2 cells, and lipoxin A_4_ inhibits inflammatory responses by these cells (128, 129). Apart from this, lipoxin A_4_ also binds to the CysLT_1_R with equal affinity as LTD_4_ and acts as an antagonist by regulating the action of LTD_4_ as well as leukocyte trafficking (130).

A characteristic feature of N-ERD is the diminished lipoxin levels, although the mechanism accounting for this remains unclear (117). At baseline, N-ERD patients have a lower level of lipoxins in plasma and blood leukocytes as compared to aspirin tolerant asthmatic patients (131, 132). The levels of lipoxins also correlate directly with the severity of asthma. Studies comparing N-ERD and aspirin tolerant asthma patients suggest a link between aspirin sensitivity and the ability to generate lipoxins (117). This may potentially involve altered metabolism of the lipoxin precursor 15(S)-HETE (133, 134). The low levels of lipoxins in N-ERD could be due to preferential conversion of 15(S)-HETE into 15-oxo-Eicosatetraenoic acid (15-oxo-ETE) rather than lipoxins (135). Another possible cause for reduced lipoxins in N-ERD could be the low levels of PGE_2_, since PGE_2_ is capable of switching lipid mediator biosynthesis from LTB_4_, a proinflammatory product of 5-LO, to the production of 15-LO product, lipoxin A_4_ (136). Additionally, there is a reduced expression of the ALX/FPR2 receptor genes in the peripheral blood of severe asthma patients, which included NERD patients, likely leading to an overall reduction in lipoxin function (137).

Another aspect of lipoxin biosynthesis that may be relevant to N-ERD is the finding that aspirin has different effects on the COX-1 and COX-2 enzymes, which leads to generation of bioactive anti-inflammatory tetraene eicosanoids known as aspirin-triggered 15-epi-lipoxins (138). Aspirin covalently modifies both COX-1 and COX-2 by acetylating their active site serine residues (139). Acetylation of COX-1 results in the complete inhibition of its activity of generating prostaglandins from arachidonic acid (1, 140). In contrast, the acetylation of a serine residue in COX-2 results in a switch from a prostaglandin-producing COX-2 into an enzyme that converts arachidonic acid to the R stereoisomer of 15-HETE (i.e., 15(R)-HETE) (138, 140). 15(R)-HETE is then metabolized in a transcellular manner by 5-LO in leukocytes to produce 15-epi-lipoxin. A defect in this aspirin-dependent pathway in N-ERD patients might be another mechanism contributing to their deficiency in lipoxins.

## Treatment of N-ERD

There are several treatments that benefit N-ERD patients, including leukotriene modifiers, corticosteroids, anti-immunoglobulin E (IgE) monoclonal antibody, endoscopic sinus surgery, and aspirin desensitization and maintenance therapy (141). In this section we will focus on the beneficial effects of aspirin treatment in N-ERD and discuss possible mechanisms for its action.

### Aspirin Desensitization and Maintenance Therapy

Although aspirin acts as a potent and rapid trigger of respiratory symptoms in N-ERD patients, extensive clinical experience shows that desensitization can be achieved in many patients and reinforced by continuous aspirin maintenance therapy. One of the earliest reports on aspirin benefits in the treatment of N-ERD was conducted in 1980 by Stevenson et al. They showed that aspirin-sensitive patients were able to continue on a daily dose of aspirin with subsequent symptom improvement and a decrease in daily corticosteroid dose (142). Since then, various short and long-term studies have been conducted that corroborate the benefits of aspirin desensitization followed by aspirin maintenance therapy in N-ERD (22, 143, 144). Aspirin desensitization in N-ERD involves a gradual administration of aspirin to the patient in a controlled setting. Available protocols suggest administration of 40 mg, 80, mg, 160, mg, and 325 mg every 60 to 90 minutes, until the patient is able to tolerate a dose of 325 mg of aspirin. Following aspirin desensitization, aspirin maintenance therapy is initiated, commonly with twice daily dose of 650 mg followed by tapering to 325 mg twice daily (145, 146). Clinically, N-ERD patients treated with aspirin maintenance regimens experience a reduction in their daily need for maintenance corticosteroid, improvement in clinical symptoms of asthma, and experience a significant reduction in ER visits or hospitalization due to asthma (16, 20, 147, 148). Endoscopic sinus surgery enhances the response to aspirin treatment, and can even create responses to aspirin maintenance in cases that previously failed aspirin treatment (9, 149). The effects of NSAIDs other than aspirin in alleviating the symptoms of N-ERD have not been extensively studied. At present, a combination of endoscopic sinus surgery followed by aspirin desensitization and aspirin maintenance therapy is widely used to treat N-ERD patients.

## Potential Mechanism of Aspirin Action

**Figure 1:**
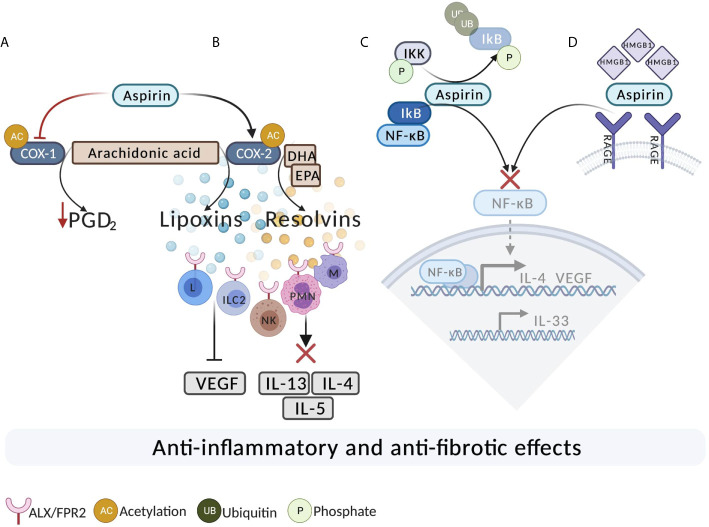
The potential mechanisms of aspirin action in N-ERD. **(A)** Aspirin acetylates and completely inhibits COX-1. As a result of long-term, high-dose aspirin treatment, there is a reduction in the level of PGD_2_. **(B)** Aspirin acetylates COX-2. Acetylation of COX-2 results in the production of 15(R)-HETE, leading to generation of 15-epi-lipoxins from arachidonic acid and resolvins from DHA and EPA. Lipoxins and resolvins bind to ALX/FPR2 on ILC2, T, B, and NK cells leading to decreased inflammation. Lipoxins also exert antifibrotic effects by reducing the expression of VEGF.  Resolvins act on ALX/FPR2 on macrophages and PMN and promote the resolution of the allergic reaction. **(C)** Aspirin may inhibit the activation of NF-κB. Aspirin potentially binds to IKK-β (IκB Kinase) and inhibits the degradation of IκB, resulting in inhibition of the NF-κB pathway.  **(D) **Inhibition of high mobility group box 1 protein (HMGB1). Aspirin directly binds and inhibits HMGB1 and subsequently leads to the inhibition of the downstream pro-inflammatory activating signaling pathway *via* NF-ĸB. DHA, Docosahexaenoic acid; EPA, Eicosapentaenoic acid; ILC2, innate lymphoid type 2 cells; M, macrophage; L, lymphocyte; NK. Natural Killer; PMN, Polymorphonuclear; UB, ubiquitin; P, Phosphate.

The underlying mechanism of aspirin’s anti-inflammatory action in N-ERD is not completely understood. Long-term aspirin treatment has a beneficial effect in a wide range of diseases. Its therapeutic effects are well-established in cardiovascular disease, thalassemia, and various types of cancers with particular relevance to prevention of colorectal cancers (150–152). **Table 1** summarizes the function of aspirin in different diseases. There is a suggestion that the continuous blocking of the COX-activity is generally associated with aspirin treatment benefits (18, 53, 176). Since aspirin is a powerful inhibitor of COX-1, long-term aspirin action has resulted in reduced levels of prostaglandin D_2_ (75, 177). However, there are difficulties in explaining the beneficial effects of aspirin maintenance therapy in N-ERD by solely considering known effects on the arachidonic acid pathway. For example, a potential pathway for alleviating the symptoms of N-ERD should include lowering urinary levels of LTE_4_ as a measure of cysLTs and increasing the levels of PGE_2_. However, high doses of aspirin treatment of N-ERD patients for eight weeks was found to either not change or increase urinary LTE_4_ levels (75, 177), and also induced a further reduction in urinary PGE-metabolite levels. Thus, aspirin therapy appears to reduce nasal symptoms in N-ERD patients without correcting any of the known defects in PGE_2_ and cysLT production. These findings imply involvement of other pathways that may be unrelated to direct COX-1/2 inhibition and the modulation of products of the cyclooxygenase and lipoxygenase pathways. Some of the possible alternative mechanisms are summarized in **Figure 1** and are discussed in the sections that follow.

**Table 1:** Effect of Aspirin on various diseases.

Diseases	Functions	Applications
Cardio-vascular diseases (152–160)	Controls cardiac hypertrophy and fibrosis by modulating angiotensin, thromboxane, and prostacyclin production, inhibits platelets activation and aggregation. Downregulates NF-κB, VCAM-1 and oxygen free radicals leading to reduction of vascular inflammation *via* p38MAPKs-NF-κB-VCAM-1 pathway.	Improves vascular dysfunction, cardiac hypertrophy, and oxidative stress. Reduces risk of non-fatal myocardial infarction. A low dose of aspirin may prevent from developing cardiovascular diseases, prevents second heart attack/stroke. Reduces stroke chances in diabetic patient with or without a history of heart disease. Prevents myocardial infarction and decreases incidence of stent thrombosis in patients with atrial fibrillation and atherosclerotic cardiovascular disease.
Thalassemia (161, 162)	Downregulates CD40L expression leading to reduction in inflammation and thrombosis in patient with thalassemia and β-thalassemia major.	Prevention of thrombosis and protect from new white matter brain lesions in beta thalassemia major patients.
Tumorigeneses of hepatic, ovarian and colon cancer (163–165)	Downregulates bcl-2 expression and upregulating Bax and p53 to inhibit tumorigenesis in lung, ovarian and colon cancer cells. Downregulates MMP-2 and E-cadherin expression along with platelet activation resulting in reduced invasion of hepatic adenocarcinoma cell line.	Reduces tumor growth and metastasis, inhibits tumor cell invasion.
Colorectal and colon cancers (166)	Inhibits WNT and MAPK pathways, arrest cell cycle.	Induces cancer cell apoptosis.
Hepatocellular carcinoma (167, 168)	Modulates NF-κB/P4HA2 axis and LMCD1-AS1/let-7g/P4HA2 axis in hepatocellular carcinoma. Induces high expression of DNA mismatch repair proteins hMLH1, hMSH2, hMSH6 and hPMS2.	Inhibits hepatocellular carcinoma. Inhibits cell cycle and induces apoptosis of human colon cancer cells.
Esophageal, prostate, breast, gastric, and gastro-intestinal cancers (153, 169–173)	Downregulates atherothrombosis, inactivates platelet aggregation and cancer metastasis in esophageal and gastro-intestinal cancers. Inhibits angiogenesis in gastric cancer. Enhances nitric oxide production leading to IKKβ-mediated inhibition of NF-κB activity in gastric, prostate and breast cancer stem cells.	Reduces risk of esophageal, gastric, breast, prostate, and gastro-intestinal cancers.
Bone degeneration (174)	Activation of osteoblastic bone formation and inhibition of osteoclast activities *via* cyclooxygenase-independent *via* Wnt/β-catenin pathways.	Maintaining bone mass, qualities, bone self-regeneration, and fracture-healing.
Non-alcoholic fatty liver disease (175)	Inhibits lipid biosynthesis, decreases levels of TNF-α and angiotensin II type1 receptor along with activation of PPAR*δ*-AMPK-PGC-1*α* pathway, as well as by modulating the mannose receptor and C-C chemokine receptor type 2 levels in macrophages.	Improves non-alcoholic fatty liver disease and atherosclerosis.

### Aspirin-Triggered Lipoxins and Inhibition of Growth Factors

Lipoxins and 15-epi-lipoxin reduce symptoms of severe asthma, airway inflammation and airway hyper-responsiveness (119, 120, 178, 179). For example, lipoxin A_4_ blocks airway hyper-responsiveness and pulmonary inflammation by decreasing leukocytes and mediators such as interleukin-5 (IL-5) and interleukin-13 (IL-13) (120). 15(S)-HETE is considered to function both as a pro- and an anti-inflammatory molecule and is a precursor to lipoxins and 15-epi-lipoxin. Kowalski et al. showed for the first time with cultured epithelial cells from nasal polyps, a significant increase in 15(R)-HETE generation following aspirin exposure was observed only in cells derived from N-ERD patients (87). This was closely followed by another study which showed a similar observation in peripheral blood leukocytes where higher levels of 15(R)-HETE were generated in N-ERD patients upon aspirin exposure, when compared to the aspirin tolerant asthma patients (132). In addition, a recent report showed that N-ERD patients with higher baseline 15(S)-HETE plasma levels showed a greater improvement in respiratory symptoms and pulmonary function during treatment with aspirin (134). Thus, aspirin treatment benefit in N-ERD patients may be associated with the ability to convert 15(R)-HETE to 15-epi-lipoxin by 5-lipoxygenase.

Lipoxins, including aspirin-triggered lipoxins, may potentially exert antifibrotic effects by reducing the expression of vascular endothelial growth factor (VEGF). VEGF causes angiogenesis and drives the proliferation and survival of the epithelial cells in chronic rhinosinusitis (180, 181). An increased level of VEGF is also found in the nasal lavage of chronic rhinosinusitis patients with nasal polyposis (181). The increased PGD_2_ levels in nasal polyps of patients with chronic rhinosinusitis has been identified as a dominant factor in inducing the production of VEGF *via* the DP receptors, and an increased level of VEGF has been documented in nasal polyps of N-ERD patients (24, 182, 183). In N-ERD, overexpression of PGD_2_ may explain the increase in VEGF, and suppression of VEGF production could be a potential mechanism, by which nasal polyp recurrence is reduced in N-ERD. While the effect of aspirin on VEGF expression levels has not been studied in N-ERD, its effect in other disorders has been documented. For example, aspirin inhibits tumor angiogenesis and cell proliferation by reducing the expression of VEGF (184, 185). Lipoxin A_4_ suppresses tumor growth by inhibiting the VEGF production in a hepatocarcinoma cell line (186). Even though the direct effects of aspirin to reduce the levels of VEGF has not been studied in N-ERD, it could be a possible mechanism of aspirin action.

### Production of Resolvins and Aspirin-Triggered Resolvins

The enzymatic oxygenation of arachidonic acid, an omega-6 fatty acid, produces both anti- and pro-inflammatory lipid mediators. On the other hand, metabolism of the omega-3 fatty acids such as eicosapentaenoic acid (EPA) and docosahexaenoic acid (DHA) mainly produces pro-resolving mediators such as resolvins of the E-series and D-series and protectins. Resolvin D1 (RvD1) and resolvin E1 (RvE1) promote resolution of inflammation in allergic airway disease, and have also been implicated in resolution of inflammation in tumors (187, 188). Resolvin D2 (RvD2) and resolvin D3 (RvD3) are also known to have potent inflammation resolving activities (188, 189). DHA is converted to the D-series resolvins *via* the sequential oxygenation initiated by 15-LO and followed by 5-LO *via* 17S-H(p)DHA (17(S)-hydroperoxy-docosahexaenoic acid). On the other hand, aspirin acetylated COX-2 and 5-LO converts DHA to aspirin triggered resolvins *via* 17R-H(p)DHA (17(R)-hydroperoxy-docosahexaenoic acid) (190). Along with the D-series resolvins, their aspirin-triggered counterparts also function as anti-inflammatory and pro-resolving factors in tumors and allergic airway inflammation by reducing polymorphonuclear leukocyte infiltration (189, 191).

RvD1 and its aspirin-triggered 17R epimer (AT-RvD1) promote resolution in allergic airway inflammation by decreasing eosinophil counts and proinflammatory mediators, and by increasing the clearance of allergens from the bronchial tree (192). These molecules can also reduce the levels of Th2 cytokines IL-4 and IL-13 in the lung, as measured in bronchoalveolar lavage fluids (192). Both RvD1 and aspirin-triggered RvD1 exert their pro-resolving effects *via* the ALX/FPR2 receptors that are also used by lipoxins to exert their anti-inflammatory actions (193, 194). While the effects of resolvins in N-ERD have not been studied extensively, indirect evidence of their relevance is suggested by the finding that a diet high in omega-3 fatty acids can improve N-ERD-associated symptoms, and this is associated with increased levels of RvD1 and reduced urinary levels of pro-inflammatory LTE_4_ and PGD-metabolite (195). Enhanced production of resolvins upon aspirin treatment should thus be examined further as a possible mechanism of aspirin benefits in N-ERD.

### Aspirin Effects on Inflammatory Cytokine Levels

Cytokines play an integral part in allergic inflammation. IL-4 for example, is involved in the progression of several allergic conditions (196, 197). In N-ERD, overexpression of IL-4 in the nasal and sinus mucosa of the nasal polyps contributes to the pathogenesis of the disease (198). IL-4 can strongly and selectively upregulate the expression of the LTC_4_S mRNA and proteins (199). It can also upregulate the m-RNA and protein expression of CysLT_1_R (an LTD_4_ receptor) in human monocytes and monocyte-derived macrophages, thus also contributing to the pathogenesis of allergic disease and asthma by modulating the responsiveness to LTD_4_ (54). A possible mechanism is the activation of the transcription factor signal transducer and activator of transcription 6 (STAT6) by IL-4 (200). At the transcriptional level, the expression of CysLT_1_R and LTC_4_S can be induced by IL-4 through a STAT6 response element located in the promoter region of CysLT1R and LTC_4_S (201, 202).

Prolonged aspirin use reduces the level of IL-4 in sputum samples of N-ERD patients (18, 144, 203, 204). The mechanism by which aspirin reduces IL-4 levels in N-ERD is not well understood, but one possibility could be that this occurs at the level of IL-4 transcription as observed in an *in vitro* analysis where aspirin (1 mM) was effective in inhibiting gene expression of IL-4 in activated CD4^+^ T cells (198, 204). This aspirin-induced reduction in the levels of IL-4 transcription can lead to beneficial downstream effects. For example, an aspirin-induced reduction in the level of IL-4 can prevent the activation of STAT6 as seen in *in vitro* studies in human PBMCs (205). In case of N-ERD, two studies have shown that aspirin can inhibit the IL-4-STAT6 axis, thus resulting in therapeutic benefits associated with aspirin treatment by reducing the level of CysLT_1_R and LTC_4_S (202, 206).

### Regulation of the NF-κB Pathway

Nuclear factor kappa light chain enhancer of activated B cells (NF-κB) is a protein complex that plays an important role in promoting inflammation, cell proliferation and survival. It is present in complex with inhibitor of nuclear factor κB (IκB), an inhibitory protein. The IκB Kinase (IKK) is an enzyme complex consisting of two catalytic subunits involving kinases (IKK-α and IKK-β) and a regulatory subunit (IKK-γ). Upon activation, IKK phosphorylates IκB resulting in the degradation of the protein complex and translocation of NF-κB to the nucleus. After its own activation, NF-κB can activate the transcription of various pro-inflammatory cytokines, chemokines and adhesion molecules. In cancer, a deregulated NF-κB pathway promotes tumor cell survival, proliferation, migration, invasion, angiogenesis, and resistance to therapy (169).

Increased expression of NF-κB is associated with nasal polyposis (207). The involvement of the NF-κB signaling pathway has been well documented in chronic airway diseases such as asthma by activation and translocation of NF-κB *via* the phosphorylation of IKK-β (208, 209). Interleukin-25 (IL-25) induces myofibroblast differentiation, extracellular matrix production and matrix metalloprotease expression in nasal fibroblasts *via* the NF- κB signaling pathway, thus aiding the process of the tissue growth and leading to nasal polyposis in chronic rhinosinusitis (210). IL-25 is also upregulated in N-ERD but, to our knowledge, no direct association of the NF-κB pathway has been established in case of N-ERD. An elevated level of the ligand for Receptor Activator of NF- κB (RANK-L) was observed in the tissue homogenates from nasal polyps of N-ERD patients (211). An increased expression of RANK-L could result in downstream activation of NF- κB, however this will have to be investigated further to determine the exact role of NF- κB in N-ERD.

There is evidence that, besides inhibiting the cyclooxygenase-prostaglandin axis, aspirin also mediates anti-tumor and anti-inflammatory effects through inhibiting NF-ĸB (212). There are various ways by which aspirin can inhibit NF-κB, which may include aspirin binding to the kinase IKK-β to reduce its accessibility to ATP and prevent phosphorylation of IκB (212, 213). Stabilization of IκB in this way has been suggested as a possible mechanism of inhibition of allergic airway inflammation by aspirin (192). Resolvin D1 and aspirin triggered resolvin D1 can reduce airway eosinophilia by decreasing IκB-α degradation, leading to a reduced activation of NF-ĸB (192). Therefore aspirin can eventually reduce the activation of NF-ĸB *via* an indirect mechanism involving resolvins, although the exact mechanism remains unknown at this time. Association of NF-κB with nasal polyp formation in N-ERD needs further investigation as a potential target for anti-inflammatory aspirin action.

### Inhibition of High Mobility Group Box 1 Protein

High mobility group box 1 or HMGB1 is a non-histone, chromatin binding protein that belongs to the family of damage-associated molecular patterns and plays an important part in inflammation. HMGB1 is involved in cancer angiogenesis and in cancer progression and metastasis development (214, 215). It exerts its pro-inflammatory and pro-angiogenic activity *via* its interaction with various toll-like receptors but most importantly toll-like receptor 4 and *via* receptor for advanced glycation end products (RAGE) (216, 217). These interactions then activate downstream signaling pathways such as MAPK and NF-ĸB which in turn releases pro-inflammatory cytokines such as tumor necrosis factor-α, interleukin-1,-6,-8, and VEGF (218). Apart from cancer, HMGB1 is also involved in asthma pathogenesis as HMGB1 and its receptor RAGE are overexpressed in the sputum of severe asthma patients (219). The protein-receptor pair is also overexpressed in the nasal tissues of those diagnosed with eosinophilic chronic rhinosinusitis with nasal polyps (220, 221). Interleukin-33 (IL-33) is a an inducer of mast cell activation and innate type 2 immunity and is present in increased levels in the nasal polyp tissues of patients with N-ERD (222). A recent study in mice showed that stimulation with LTC_4_ upregulates the expression of surface HMGB1 (223). HMGB1 can then signal through RAGE resulting in the amplification of CysLT_2_R-mediated platelet activation which can lead to an increase in IL-33 levels.

Due to its pro-inflammatory effects, HMGB1 is an important drug target and a potential biomarker for various neurodegenerative diseases and cancers. It has been suggested that aspirin is capable of inhibiting HMGB1-mediated tumor progression (224). Even though the exact mechanism of HMGB1 inhibition by aspirin is not fully known, aspirin may function by directly binding to HMGB1 and suppressing its proinflammatory functions even at low doses (225). HMGB1 activates VEGF, downstream signaling of NF-ĸB pathway, and IL-33. Inhibition of HMGB1 by aspirin can therefore lead to a downregulation of these pathways, although a clinical relevance of these potential actions in N-ERD needs to be further investigated.

### Epigenetic Changes

Aspirin’s effectiveness in treatment of cancer may be related to epigenetic changes, such as histone acetylation. Histone acetylation involves histone acetyltransferase and histone deacetylase enzymes (226), and the activities of these enzymes can be regulated by aspirin (163). In fact, inhibition of histone deacetylase in general is now considered to be a possible therapeutic approach for colorectal and other cancers by regulating epigenetic changes (227–229). Aspirin in particular was shown to modulate epigenetic changes in colon cancer by inhibiting the activity of histone deacetylase which leads to restoration of histone 3 lysine 27 acetylation, eventually suppressing levels of tumor necrosis factor-α and interleukin-6 (230).

Epigenetic changes such as hyper and hypo-methylation of genes mainly associated with the arachidonic acid pathway have been observed in N-ERD (231). In the nasal polyp samples of N-ERD patients, Cheong et al. observed that the following genes were hypomethylated: the *PGDS* gene encoding prostaglandin D synthase, the *ALOX5AP* gene encoding 5-LO activation protein, and the *LTB4R* gene encoding the leukotriene B4 receptor, while the *PTGES* gene encoding prostaglandin E synthase was hypermethylated (231). Laidlaw et al. also observed epigenetic changes in the form of histone acetylation at the *PTGER2* promoter that dysregulates the EP_2_ receptor expression in the nasal fibroblast of N-ERD patients (103). Whether or not these epigenetic changes could function as possible targets for aspirin therapy in N-ERD is still unknown.

## Conclusion

### Therapeutic Implications

Aspirin desensitization and maintenance therapy following endoscopic sinus surgery is an effective treatment for N-ERD patients that reduces the recurrence of nasal polyps. Aspirin and its use as an anti-inflammatory agent have been widely studied in cancer, but the exact mechanism of its actions in N-ERD is not completely understood. It is also unexplained why some N-ERD patients fail aspirin desensitization and maintenance therapy. In this review we summarized the potential pathways of aspirin action based on the available knowledge of aspirin’s effectiveness on eicosanoids, growth factors, pro- and anti-inflammatory pathways, and cytokines. Exploring these mechanisms may highlight the diversity of aspirin action. Mechanistic studies may help in developing new and more effective treatments for N-ERD using aspirin and other agents. Information on aspirin actions may help identify N-ERD patients who are appropriate candidates for this effective and affordable treatment.

### Limitations and Future Directions

The mechanism of aspirin action to relieve the symptoms of N-ERD is not fully understood. There are various avenues for further investigation of aspirin actions in N-ERD. A major emphasis has been primarily on the arachidonic acid pathway and its regulation by aspirin in N-ERD. Aspirin exerts its anti-inflammatory effects in other diseases, for example in cancer, by several other pathways. Limited information is available for some of these pathways such as production of resolvins and modulation of epigenetic changes in N-ERD. Further studies are needed to determine the role of these pathways in N-ERD. The role of NF-κB, VEGF, HMGB1, and RAGE in N-ERD needs to be better defined. Long-term studies with an emphasis on molecular pathways would provide a robust understanding of the disease and pathways that could serve as therapeutic targets.

## Author Contributions

ES, MA, and EJ were involved in conceptualizing the review. All authors contributed to the drafting and editing of the manuscript. All authors contributed to the article and approved the submitted version.

## Funding

This work was supported by grant R21AI146804 from the National Institutes of Health (NIH)/National institute of Allergy and Infectious diseases (NIAID). Portions of this work were supported by the generous contribution of Hiram and Jeanne Gray Endowment Fund. Its contents are solely the responsibility of the authors and do not necessarily represent the official views of the NIH.

## Conflict of Interest

EJ served on the Advisory Board for GSK, Sanofi/Regeneron, and Novartis/Genentech; she is a consultant for GSK and has research support from AstraZeneca and from Cumberland Pharmaceuticals.

The remaining authors declare that the research was conducted in the absence of any commercial or financial relationships that could be construed as a potential conflict of interest.
